# Pediatric malignancies presenting as a possible infectious disease

**DOI:** 10.1186/1471-2334-7-44

**Published:** 2007-05-22

**Authors:** Sarah E Forgie, Joan L Robinson

**Affiliations:** 1 Department of Pediatrics and Stollery Children's Hospital, Edmonton, Alberta, Canada

## Abstract

**Background:**

The clinical, laboratory, and radiological features of malignancy can overlap with those of infection. The purpose of this study was to determine the findings in children who were initially thought to have an infectious disease but ultimately proved to have a malignancy.

**Methods:**

The database of patients diagnosed with a malignancy in the Northern Alberta Children's Cancer Program (NACCP) January 1, 1993 to December 31, 2003 was merged with the database of inpatients referred to the infectious diseases service at the Stollery Children's Hospital and charts were reviewed on all patients referred to the infectious diseases consult service prior to the diagnosis of malignancy.

**Results:**

An infectious diseases consultation for diagnosis was requested in 21 of 561 patients prior to the confirmation of malignancy, and 3 of these 21 patients had both infection and malignancy (leukemia (N = 13), lymphoma (N = 3), rhabdomyosarcoma (N = 1), Langerhan's cell histiocytosis (N = 1), fibrous histicocytosis (N = 1), ependymoma (N = 1), and neuroblastoma (N = 1). The most common reason for infectious diseases consultation was suspected muskuloskeletal infection (N = 9). A palpable or radiographically enlarged spleen was noted in 11 patients (52%). All but 2 patients had abnormal hematologic parameters while an elevated lactate dehydrogenase (LDH) occurred in 10 patients (48%). Delay of diagnosis because of investigation or therapy for an infectious disease occurred in only 2 patients.

**Conclusion:**

It is not common for treatment of pediatric malignancies to be delayed because infection is thought to be the primary diagnosis. However, pediatric infectious diseases physicians should consider malignancy in the differential diagnosis when they see patients with fever and bone pain, unexplained splenomegaly or abnormal complete blood cell counts. Other clues may include hepatomegaly or elevated LDH.

## Background

Approximately 1300 children develop cancer each year in Canada. [1] The age standardized incidence rates in Canada have been relatively constant over the past 15 years, and in Alberta, the numbers are similar to national data at 15.13/100,000 children under the age of 19 years. [2] Unfortunately, there can be delays in the diagnosis because the signs and symptoms of malignancy may be initially misdiagnosed as infection. The delay in diagnosis and therapy may increase parental anxiety and ultimately adversely affect outcome. [3-5]

There have been no previous systematic assessments of the clinical findings, laboratory findings and radiological findings of children initially thought to have an infection who ultimately were diagnosed with a malignancy. We hypothesized that if children were misdiagnosed with an infection initially, there would be a delay in cancer therapy. We also hypothesized that if these patients had consistent findings, this information could be used to help pediatric infectious diseases physicians know when to consider malignancy in the differential diagnosis.

## Methods

The Stollery Children's Hospital (SCH) is a 133 bed, tertiary care facility in Edmonton, Alberta, Canada that admits children from three provinces and one territory – an area covering 440,000 square miles. The Northern Alberta Children's Cancer Program (NACCP) and the pediatric infectious diseases service are the sole providers of oncologic and infectious diseases consultations respectively for children at the SCH.

The database of patients diagnosed with a malignancy in the Northern Alberta Children's Cancer Program (NACCP) January 1, 1993 to December 31, 2003 was matched with the database of inpatients referred to the infectious diseases service at the Stollery Children's Hospital within six months prior to diagnosis. Children were included in the study if the symptoms or signs resulting in the referral ultimately proved to be due to malignancy.

Inpatient charts were reviewed to determine the reason for the consultation, the timing of the consultation relative to diagnosis, the degree of suspicion of a malignancy at the time of the consultation, the type and duration of symptoms at the time of consultation (possible or documented fever, bone pain, fatigue, weight loss, or headache), the physical signs recorded at the time of the consultation (hepatomegaly defined as a liver more than 1 cm below the costal margin, splenomegaly defined as a palpable spleen, lymphadenopathy defined as a single node > 2 cm in diameter or diffuse enlargement of multiple nodes, rash, or signs of a muskuloskeletal infection) and the results of laboratory investigations (complete blood count (CBC) with normals for age being used and the differential being considered to be abnormal if there were any myeloid cells or blasts or an increased number of bands, aspartate aminotransferase (AST) (a liver enzyme), alanine aminotransferase (ALT) and lactate dehydrogenase (LDH)) and radiologic investigations. Patients were excluded if the reason for the infectious diseases consultation was not for diagnosis of the primary problem. The type of malignancy and the results of bone marrow aspiration or alternative diagnostic tests were recorded. It was then determined if delay in diagnosis occurred because an infectious disease was considered to be the most likely diagnosis.

## Results

There were 561 patients diagnosed with a malignancy by the NACCP during the study period. In 22 cases, the patient was seen by the infectious diseases service a median of 7 days prior to diagnosis (range 0 to 32 days). One patient was excluded from the study as the reason for the consultation was infection control issues related to varicella. The primary reason for the consultation in the other 21 cases was possible musculoskeletal infection (N = 9), fever with or without other symptoms (N = 4), possible respiratory infection (N = 2), possible soft tissue infection, (N = 2), splenomegaly with lymphadenopathy (N = 1), leukocytosis (N = 2), and possible cysticercosis based on imaging of the head (N = 1) (Table 1). Malignancy was not suspected by the referring physician or the infectious diseases consultant at the time of the consultation in the two cases with possible soft tissue infection (a fibrous histiocytoma presented as parietal swelling and a rhabdomyosarcoma presented as a parapharyngeal mass) and in one case of leukemia that presented as septic arthritis of the elbow. Malignancy was thought to be the likely diagnosis by both the referring physician and the infectious diseases consultant in 9 cases and was considered by either the referring physician or the infectious diseases consultant at the time of consultation in the other 9 cases.

**Table 1:** Clinical and laboratory features of 21 children with malignancies with infectious diseases consultations prior to diagnosis

**Patient**	**Age (yrs) Sex (M/F)**	**Main features at time of ID consult**	**DOS**	**Fever (days)**	**Bone pain (days)**	**Fatigue (days)**	**Weight loss (kg)**	**Headache**	**HMG (cm below CM)**	**SMG (cm below CM)**	**LN**	**Hb**	**WBC**	**Diff**	**Plt**	**LDH**	**Malignancy/Diagnostic Test**
1	1.6 F	swollen elbow, pancytopenia	high	Y(28)	No	No	No	No	Y (4)	Y (NR)	No	63	N	A	6	1015	ALL/BMA
2	12.2 M	hip and calf pain, poor appetite, fever, bruising	high	Y(7)	No	Y (21)	Y (5)	Y	No	No	No	52	14.9	A	19	869	AML/BMA
3	0.25 F	respiratory distress, shock, leukocytosis	low	No	No	No	No	No	Y (6)	Y (1)	No	N	53	A	30	3204	JMML/skin biopsy
4	14.5 F	cough, cavitary lesions on CXR	low	Y(NR)	No	No	Y (5)	No	No	Y (1)	Y	108	22.3	N	H(615)	N	Hodgkin's/lung and mediastinal node biopsies
5	1.5 F	back pain with abnormal MRI	high	Y	No	No	No	No	No	Y (1)	No	108	N	N	N	N	Langerhan's cell histiocytosis/vertebral aspirate
6	4.7 M	fever, vomiting, abdominal pain, inguinal lymphadenopathy	low	Y(5)	No	No	No	No	No	Y (CT)	Y	107	N	N	N	N	Lymphoma/node biopsy
7	2.3 M	splenomegaly, lymphadenopathy, abnormal CBC	high	No	No	No	No	No	No	Y (3)	Y	77	30.3	A	67	N	JMML/BMA
8	8.6 M	parietal swelling with imaging showing possible abscess	none	No	No	Y (70)	No	No	No	No	No	113	12.6	N	H(413)	NR	fibrous histiocytoma/dural biopsy
9	1.6 M	leukocytosis on CBC done for irritability and asthma	high	No	No	No	No	No	Y (U/S)	Y (U/S)	No	84	51.3	A	106	438	JCML/BMA
10	3.3 F	fever, lower GI bleed, pancytopenia	high	Y (7)	No	No	No	No	No	No	No	20	1.2	A	7	313	ALL/BMA
11	9.9 M	headache, blurred double vision with brain lesion on imaging	low	No	No	No	Y(5)	Y	No	No	No	N	N	N	N	N	Ependymoma/brain biopsy
12	2.2 F	fever, limp, abnormal imaging	low	Y(2)	No	No	No	No	No	No	No	106	4.4	N	N	592	Neuroblastoma/BMA
13	15. M	fever, cough, dyspnea with known pericardial and pleural effusions	low	Y(2)	No	No	No	No	No	No	No	125	58.8	A	N	N	CML/BMA
14	7.2 M	parapharyngeal mass	none	No	No	No	No	No	No	No	No	N	N	N	N	N	Rhabdomyosarcoma/biopsy of parapharyngeal mass
15	12.9 F	swollen elbow	low	Y(150)	Y(30)	No	No	No	No	Y(NR)	No	90	1.5	N	N	N	ALL/BMA
16	5. M	fever, neutropenia	high	Y(11)	No	Y(11)	Y	Y	No	Y(tip)	No	60	1.6	A	35	733	ALL/BMA
17	11.8 M	inability to weight-bear, abdominal pain	high	Y	Y(120)	Y(120)	Y	No	Y(10)	Y(1)	No	117	3.5	A	115	880	ALL/BMA
18	14.6 M	fever, myalgia, back and leg pain	low	Y(40)	Y(60)	Y(60)	Y(10)	Y	No	No	No	103	N	N	N	657	Lymphoma/BMA
19	14.8 F	fever, cellulitic rash, pancytopenia	high	Y(2)	Y	Y(NR)	No	No	Y (1.5)	No	No	87	0.8	A	18	N	ALL/BMA
20	2.8 M	limp, back pain, refusal to run	none	Y(2)	Y(7)	No	No	Y	Y(U/S)	Y(U/S)	No	71	4.5	A	83	484	ALL/BMA
21	11.8 M	back pain, fever	low	Y(5)	Y(2)	No	Y(5)	Y	No	No	No	67	N	N	N	378	ALL/BMA

A – abnormal; ALL – acute lymphocytic leukemia; AML – acute myeloid leukemia; BMA – bone marrow aspirate; cm – centimeters; CM – costal margin; CML – chronic myelogenous leukemia; CT – detected by computer tomography but not clinically; Diff – differential; DOS – degree of suspicion of malignancy at time of infectious diseases consultation; Hb – hemoglobin; HMG – hepatomegaly; ID – infectious diseases; JCML – juvenile chronic myeloid leukemia; JMML – juvenile myelomonocytic leukemia; kg – kilograms; LDH – lactate dehydrogenase; LN – lymphadenopathy; N – normal; NR – not reported; Plt – platelet; SMG – splenomegaly; U/S – detected by ultrasound but not clinically; WBC – white blood cell count; Y – yes

The type of malignancy was leukemia (N = 13), lymphoma (N = 3), rhabdomyosarcoma (N = 1), Langerhan's cell histiocytosis (N = 1), fibrous histicocytosis (N = 1), ependymoma (N = 1), and neuroblastoma (N = 1).

Fever was reported and/or documented in 15 of the 21 cases (71%). (Table 1). Other symptoms included bone pain ranging in duration from 2 days to 4 months (N = 6), fatigue ranging in duration from 1 to 70 days (N = 6), suspected weight loss ranging up to 10 kg (N = 7), and headache (N = 6). Physical signs included hepatomegaly (N = 4), a palpable spleen (N = 8) (with hepatomegaly being documented in 2 additional patients and splenomegaly in 3 additional patients by imaging), lymphadenopathy (N = 3), and a wide variety of rashes (N = 7) (Table 1 – data on rashes not shown). Only one of the patients with suspected muskuloskeletal infection had bony tenderness (patient #17) while 3 patients had signs suggestive of septic arthritis (patients #1, 12, and 15) with patient #15 having culture-negative purulent fluid aspirated from her elbow joint. All but 2 patients had an abnormal hemoglobin, white blood cell count or platelet count (Table 1). Results of ALT and AST were minimally elevated in 1 and 4 patients respectively with the highest value being an ALT of 86 in patient #6 (data not shown in table) while LDH was elevated in 10 patients.

When examining the children diagnosed with leukemia (the most common diagnosis) (N = 13), 12 (92%) had low hemoglobin, 11 (85%) had an abnormal white blood cell differential, 10 (77%) had decreased platelets, 10 (77%) had fever, 9 (69%) had an elevated LDH, 6 (46%) had hepatomegaly, 8 (62%) had splenomegaly, 6 (46%) had a decreased white blood cell count, 4 (31%) fatigue, 4 (31%) weight loss, 4 (31%) headache and 4 (31%) had an elevated white blood cell count. Seven (54%) had a rash, 2 (5%) had an elevated AST (data not shown in table).

Delay in diagnosis by 11 days occurred in patient #15 as septic arthritis was considered to be her sole diagnosis with her anemia being ignored and her leukopenia being attributed to antibiotics. Splenomegaly was noted only several days after admission. The only other patient with a delay in diagnosis because of investigation for infection was patient #20 where diagnosis was delayed by 8 days as the clinical picture fit well with discitis. An MRI was arranged as an outpatient and again abnormal findings on the CBC were ignored. In all other cases, diagnosis of malignancy was not delayed by investigation or treatment of an infectious disease. Patient #2 had leukemia and a psoas abscess and patient #3 had leukemia and severe respiratory syncytial virus infection but the diagnoses were pursued simultaneously as they had very abnormal hematologic parameters. Patient #4 had a chronic cavitary pulmonary process but no risk factors for tuberculosis so lung biopsy was arranged immediately when the bronchoalveolar lavage was negative for acid-fast bacilli, and the 19-day delay between the infectious diseases consult and diagnosis was because two lung and one mediastinal lymph node biopsies were required before a diagnosis of Hodgkin's lymphoma was made. Although patient #8 with the parapharyngeal mass and patient #14 with the parietal swelling were initially thought to have infectious processes, imaging of the parapharyngeal mass followed by biopsy and biopsy of the dura under the parietal swelling rapidly established the diagnosis of malignancy.

## Discussion

This study shows that children with malignancies can present with signs and symptoms mimicking infection including musculoskeletal symptoms, fever, splenomegaly, lymphadenopathy, leukocytosis and/or possible cysticercosis. In almost all cases, the pursuit of an infectious diagnosis was concurrent with or closely followed by the diagnosis of malignancy. In the two cases where diagnosis was delayed because of investigation of infection, more attention to the results of the CBC might have led to an earlier diagnosis. Both children had musculoskeletal symptoms and it is not uncommon for bony malignancies to mimic infection, coexist with infection, or serve as a nidus of inflammation and irritation where subsequent malignancies develop. [6] However, in both cases, the diagnostic delay was relatively short compared to previous reports where children and adults who ultimately were diagnosed with malignancy had longstanding bony lesions that were not improving on appropriate antibiotic therapy. [7-9]

Another child who had a diagnostic delay had a chronic cavitary pulmonary process that proved to be Hodgkin's disease. Primary tuberculosis most commonly presents with lymphadenopathy and parenchymal lung changes in children, but tuberculosis should still be high on the list of differential diagnoses in an older child with cavitary lung lesions. [10] Although cavitary lesions are a rare initial manifestation of Hodgkin's disease, it should be considered in the differential once tuberculosis and other infectious etiologies are ruled out. [11-13]

There were no unexpected symptoms identified that should make an infectious diseases consultant consider malignancy. Seventy-one percent of the children in the study had fever (most of which were subsequently diagnosed with leukemia). This fits with previous studies where 61% of children with acute lymphocytic leukemia (ALL) present with fever. [14] However, fever can be a symptom of infection, inflammation, malignancy, allergy and many other entities. The mechanism of fever related to infection is very similar to that of malignancy. Bacteria, by virtue of their peptidoglycan cell walls or lipopolysaccharide act as exogenous pyrogens. When injected systemically, they will trigger monocytes and macrophages to produce pro-inflammatory cytokines (including interleukins 1 and 6 and tumor necrosis factor alpha). Tumors such as Hodgkin's lymphoma, acute leukemia, lymphoma and bone sarcomas either trigger macrophages to produce these inflammatory cytokines or produce the cytokines themselves, bypassing the early host response. These pyrogenic cytokines then cause fever by acting on the hypothalamus. [15] Although it could not be tested in the current study, it has been suggested that paraneoplastic fevers may be more responsive to non steroidal anti-inflammatory drugs than are non-neoplastic fevers, and use of the "Naprosyn test" may offer some direction towards the diagnosis of malignancy. [16,17]

About one-third of the children with malignancies in the current study had significant weight loss. [18] In adults, malignancies account for one-third of all patients presenting with unintentional weight loss. Anorexia, increased metabolism, ectopic production of hormones that signal satiety, and production of chemokines such as tumor necrosis factor can contribute to weight loss in malignancies. [19,20] Chronic infectious entities such as tuberculosis, fungal disease, parasitic disease, subacute bacterial endocarditis and human immunodeficiency virus can cause weight loss by similar mechanisms (although ectopic hormone production is less likely with infections). [21]

Similarly, about one-third of the children in the study had headache and/or fatigue. As an isolated symptom, the list of differential diagnoses for headache is broad. Infections such as otitis media and sinusitis can cause headache by blockage of sinus and Eustachian tube drainage. Viral upper respiratory tract infections can trigger interferon release which can lead to headaches and fever. Space occupying lesions – whether caused by infection or malignancy can lead to headaches by impinging on other neurological structures. [22] Fatigue as an isolated symptom in children is also very common and non-specific, but one clue to an underlying malignancy may be the persistence of the fatigue, since most of the children in this study had this symptom for almost two months (mean 56.4 days, range 11–120 days). [23,24]

Bone pain was reported in about one-third of the children. This fits with the literature where 23% of children with ALL presented with bone pain. [24] Bone pain in malignancy can be due to primary (benign or malignant) tumors of bone, metastatic bone lesions or bone marrow infiltration. Osteoid osteomas, osteosarcomas or Ewing's sarcoma are the most frequent primary bone tumors in children. Metastatic bone lesions are uncommon in pediatrics, except those secondary to neuroblastoma or Ewing's sarcoma. Bone marrow infiltration is the most common underlying mechanism of musculoskeletal pain in lymphoproliferative disorders. [25] In the current study, bone marrow infiltration was the most likely mechanism of bone pain since 80% of the children with bone pain were subsequently diagnosed with leukemia and 20% with lymphoma. There were no cases of primary bone malignancies or metastatic diseases to bone that were initially investigated as infections.

Abdominal imaging was more sensitive than physical examination with detection of hepatomegaly and splenomegaly increasing from 19% to 28% and from 38% to 52% respectively. Overall, 24% of the children in our study presented with the findings of hepatosplenomegaly together and 14% presented with lymphadenopathy. Interestingly no child with leukemia presented with lymphadenopathy on examination, which is in stark contrast to the literature where 50% of children with ALL present with lymphadenopathy. [26] It seems likely that the lack of lymphadenopathy contributed to the difficulty in making the correct diagnosis.

Laboratory tests routinely done to evaluate children with symptoms such as fever and bone pain can initially be misleading in children with malignancies. [25-27] In the current study, the results of the CBC were usually a clue to the diagnosis of malignancy. All but two patients had an abnormal hemoglobin, white blood cell count and/or platelet count. For the children with leukemia, every child had at least one abnormal hematologic parameter with 85% having an abnormal white blood cell differential. Approximately half of the patients in our study had an elevated LDH most of whom were subsequently diagnosed with leukemia. This is not surprising, since an elevated LDH can indicate rapid cell turnover – which is seen with malignancy and cell damage from other processes. [28,29] We found that other lab tests such as the liver enzymes AST and ALT were not as helpful in making the correct diagnosis. There is very little data regarding AST and ALT as screening tests for malignancies (with the obvious exception being hepatoblastoma and other primary malignancies of the liver). [30,31]

Results of this study were similar to those in a study of patients referred to rheumatology clinics who had undiagnosed malignancies, where about half had fever, fatigue, or weight loss and about one-third had hepatomegaly, abnormal CBC, or elevated LDH. [32]

The chief limitation of the study is that it was a retrospective chart review so some of the data may not have been recorded on the chart, including the fact that malignancy was suspected. Another limitation is the absence of a control group of children with malignancy not initially investigated for infection.

## Conclusion

It is not unusual for an infectious diseases consultant to be asked to review children who ultimately prove to have a malignancy. The vast majority of the time, investigation or therapy for infection does not significantly delay the diagnosis of malignancy. A history of prolonged fatigue, fever and bone pain, physical findings of hepatomegaly and/or splenomegaly, abnormalities on the CBC and an elevated LDH are all clues to the diagnosis of malignancy.

## Competing interests

The author(s) declare that they have no competing interests.

## Authors' contributions

JLR conceived of the study, designed the data collection form, reviewed the charts, analyzed the data and revised the manuscript. SEF revised the data collection form, reviewed charts and wrote the manuscript. Both authors have read and approved the final version of the manuscript.

## Pre-publication history

The pre-publication history for this paper can be accessed here:


